# Relationship between temperature and *Anopheles gambiae* sensu lato mosquitoes' susceptibility to pyrethroids and expression of metabolic enzymes

**DOI:** 10.1186/s13071-022-05273-z

**Published:** 2022-05-08

**Authors:** Thomas Peprah Agyekum, John Arko-Mensah, Paul Kingsley Botwe, Jonathan Nartey Hogarh, Ibrahim Issah, Samuel Kweku Dadzie, Duah Dwomoh, Maxwell Kelvin Billah, Thomas Robins, Julius Najah Fobil

**Affiliations:** 1 Department of Biological, Environmental and Occupational Health Sciences, School of Public Health, University of Ghana, P.O. Box L.G. 13, Accra, Ghana; 2 Department of Environmental Science, Kwame Nkrumah University of Science and Technology, Kumasi, Ghana; 3 Parasitology Department, Noguchi Memorial Institute for Medical Research (NMIMR), University of Ghana, P.O. Box LG 581, Accra, Ghana; 4 Department of Biostatistics, School of Public Health, College of Health Sciences, University of Ghana, Legon, Ghana; 5 Department of Animal Biology and Conservation Science, University of Ghana, P.O. Box L.G. 67, Accra, Ghana; 6 Department of Environmental Health Sciences, University of Michigan, 1415 Washington Heights, Ann Arbor, MI 48109 USA

**Keywords:** *Anopheles gambiae*, Bioassay, Biochemical analysis, Climate change, Insecticide, Metabolic enzyme, Susceptibility

## Abstract

**Background:**

Malaria remains one of the most devastating diseases globally, and the control of mosquitoes as the vector is mainly dependent on chemical insecticides. Elevated temperatures associated with future warmer climates could affect mosquitoes' metabolic enzyme expression and increase insecticide resistance, making vector control difficult. Understanding how mosquito rearing temperatures influence their susceptibility to insecticide and expression of metabolic enzymes could aid in the development of novel tools and strategies to control mosquitoes in a future warmer climate. This study evaluated the effects of temperature on the susceptibility of *Anopheles gambiae* sensu lato (s.l.) mosquitoes to pyrethroids and their expression of metabolic enzymes.

**Methods:**

*Anopheles gambiae* s.l. eggs obtained from laboratory-established colonies were reared under eight temperature regimes (25, 28, 30, 32, 34, 36, 38, and 40 °C). Upon adult emergence, 3- to 5-day-old female non-blood-fed mosquitoes were used for susceptibility tests following the World Health Organization (WHO) bioassay protocol. Batches of 20–25 mosquitoes from each temperature regime (25–34 °C) were exposed to two pyrethroid insecticides (0.75% permethrin and 0.05% deltamethrin). In addition, the levels of four metabolic enzymes (α-esterase, β-esterase, glutathione S-transferase [GST], and mixed-function oxidase [MFO]) were examined in mosquitoes that were not exposed and those that were exposed to pyrethroids.

**Results:**

Mortality in *An. gambiae* s.l. mosquitoes exposed to deltamethrin and permethrin decreased at temperatures above 28 °C. In addition, mosquitoes reared at higher temperatures were more resistant and had more elevated enzyme levels than those raised at low temperatures. Overall, mosquitoes that survived after being exposed to pyrethroids had higher levels of metabolic enzymes than those that were not exposed to pyrethroids.

**Conclusions:**

This study provides evidence that elevated temperatures decreased *An. gambiae* s.l. mosquitoes' susceptibility to pyrethroids and increased the expression of metabolic enzymes. This evidence suggests that elevated temperatures projected in a future warmer climate could increase mosquitoes' resistance to insecticides and complicate malaria vector control measures. This study therefore provides vital information, and suggests useful areas of future research, on the effects of temperature variability on mosquitoes that could guide vector control measures in a future warmer climate.

**Graphical Abstract:**

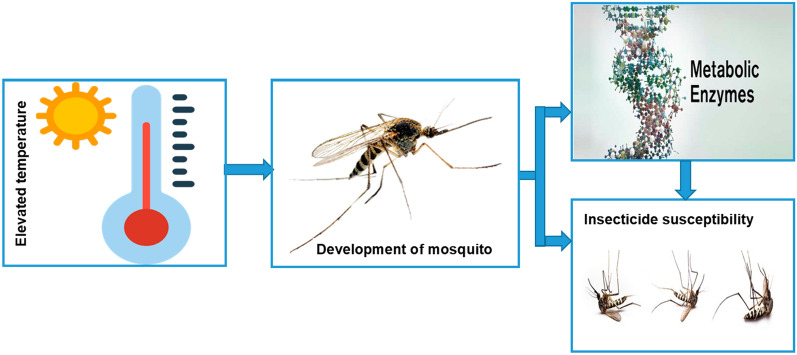

**Supplementary Information:**

The online version contains supplementary material available at 10.1186/s13071-022-05273-z.

## Background

The control of mosquito vectors using chemical insecticides remains the primary line of action to eradicate malaria [1–3]. According to the World Health Organization (WHO), six classes of insecticides—pyrethroids, organochlorines, carbamates, organophosphates, pyrroles, and phenyl pyrazoles—are used in mosquito control programs worldwide [4]. However, the development and rapid spread of insecticide resistance in mosquitoes such as *Anopheles gambiae* could affect malaria control measures. The *An. gambiae* complex consists of nine morphologically identical sibling species. They include *An. gambiae* sensu stricto (s.s.), *An. arabiensis*, *An. merus*, *An. melas*, *An. quadriannulatus*, *An. amharicus*, *An. bwambae*, *An. coluzzii*, and *An. fontenillei* [5–7]. In sub-Saharan Africa, *An. gambiae* complex is the primary vector responsible for causing malaria [8]. Hence, the resistance of *An. gambiae* mosquitoes to insecticides could threaten efforts aimed at malaria control, especially in regions such as Africa, Asia, and Latin America, where the disease is endemic and presents a significant public health threat [9–12]. Furthermore, the sustainability of the achievements made in combatting vector-borne diseases is at risk from the effects of climate change [13] as climate parameters such as temperature, rainfall, and humidity could directly affect the development and growth of mosquitoes [14, 15].

Like all poikilotherms, the biochemical and physiological processes of insects depend on temperature, which has a significant effect on insects' growth, metabolic rate, and resistance mechanisms [16–19]. In addition, temperature variations can affect insecticide susceptibility by directly modifying the physiology of mosquitoes [19]. The efficacy of insecticides against mosquitoes is, to some extent, temperature-dependent [20]. A study conducted by Whiten and Peterson [21] on the influence of ambient temperature on the susceptibility of *Aedes aegypti* mosquitoes to permethrin, a pyrethroid insecticide, found a negative correlation between temperature and mortality from 32 to 34 °C. Exposure to elevated temperatures has also been shown to increase pyrethroid resistance in mosquitoes by affecting the expression of metabolic enzymes [19].

Insects such as mosquitoes have developed various metabolic mechanisms that help them complete their life-cycles [22]. The ability of insects to degrade toxic substances is crucial to their survival, especially in the constantly changing environment [23]. Metabolic enzymes play a substantial role in the detoxification of toxic substances and the development of insecticide resistance in mosquitoes [22, 24]. The most important metabolic enzymes associated with insecticide resistance include carboxyl/cholinesterases (CCEs), cytochrome P450 monooxygenases (P450s), and glutathione S-transferases (GSTs) [25, 26]. High levels of metabolic enzymes in mosquitoes may confer resistance to different insecticide classes and detoxify insecticides before getting to the target site of action [2].

A deep dive into the literature revealed that a few studies [19, 27, 28] have examined the effects of temperature on the susceptibility of *An. funestus*, *An. arabiensis,* and *An. stephensi* but not on *An. gambiae* sensu lato (s.l.) mosquitoes (the predominant malaria vector in Ghana). In addition, only one study [19] examined the levels of metabolic enzymes in mosquitoes (cytochrome P450, α-esterase, and β-esterase) [29]. Even though these enzymes are useful in determining the metabolic resistance mechanism of mosquitoes to insecticides, they do not provide a holistic assessment of this mechanism. Available scientific evidence has reported high levels of other metabolic enzymes such as GST and acetylcholinesterase (AChE) in mosquitoes exposed to insecticides [30, 31]. However, the effects of temperature on the levels of GST and AChE in *An. gambiae* s.l. mosquitoes have not yet been investigated. Thus, evaluating the effects of rearing temperature on *An. gambiae* s.l. mosquitoes' susceptibility to insecticides and expression of metabolic enzymes could be important for developing informed malaria vector control strategies in a future warmer temperature. In this study, we evaluated how the rearing temperature of female *An. gambiae* s.l. mosquitoes affected their expression of metabolic enzymes and susceptibility to pyrethroid insecticides.

## Methods

### Experimental design

Mosquitoes were reared in climate incubators (RTOP-1000D, Zhejiang, China) at the African Regional Postgraduate Programme in Insect Science (ARPPIS), University of Ghana. Ghana has a high temperature, with average annual temperature ranging between 25 and 30 °C [32]. To forecast the effects of elevated temperatures, this study selected three temperatures within the range of 25 to 30 °C and added increments of 2 °C from 30 to 40 °C to arrive at eight temperature regimes (25, 28, 30, 32, 34, 36, 38, and 40 °C) in which mosquitoes were reared [33]. The incubators were set at 80 ± 10% relative humidity and programmed to have a photoperiod of 12:12 (light/dark) h. A HOBO MX1102 CO_2_ logger (Onset Computer Corp., Cape Cod, MA, USA) was placed in each incubator to monitor the daily temperature and relative humidity [34]. Data were downloaded daily between 10:00 and 11:00 am to ensure that temperature and humidity remained stable throughout the experiment (Additional file 1: Table S1).

### Mosquito colony

This study used two laboratory strains of *An. gambiae* s.l. mosquitoes: Tiassalé and Kisumu strains. The Tiassalé strain (a mixture of *An. gambiae* s.s. and *An. coluzzii*) is resistant to four classes of insecticides (pyrethroids, organochlorines, carbamates, organophosphates) available for malaria control [35]. It initially originated from Tiassalé, Cote d'Ivoire [35], and has been maintained in the Vestergaard-Noguchi Memorial Institute for Medical Research Vector Labs (VNVL) insectary since 2010 [36]. The Kisumu strain is a reference population (susceptible *An. gambiae*) from Kisumu, Kenya. The reference population has no history of insecticide resistance; therefore, they are susceptible to insecticides. The eggs of both strains were acquired from a colony maintained at the VNVL insectary. Larvae were fed daily on 10 mg of TetraFin goldfish flakes (Tetra Werke, Melle, Germany), and adults were provided with cotton wool soaked in a 10% sugar solution. Three to five-day-old non-blood-fed female adult mosquitoes were used in all experiments. WHO recommends using non-blood-fed mosquitoes because the physiological status of mosquitoes has a marked effect on insecticide susceptibility [4]. In addition, blood-fed mosquitoes are usually fragile, and the constituents of the blood meal could interfere with the biochemical assay [37].

### Molecular identification of *An. gambiae* s.l. mosquitoes (Tiassalé strain)

To confirm the composition of *An. gambiae* s.l., polymerase chain reaction (PCR) procedures were performed on the parent mosquitoes. One hundred adult female mosquitoes (3–5 days old, non-blood-fed) were randomly selected for molecular analysis. DNA was extracted from the sampled mosquitoes using the cetyltrimethylammonium bromide (CTAB) extraction method following the procedures described by Mouhamadou et al. [38].

Species-specific primers targeting the ribosomal DNA (rDNA) gene (intergenic spacer [IGS]) were first used to identify species of the *An. gambiae* complex using an established protocol [39]. Subsequently, short interspersed nuclear element (SINE) PCR was used for the identification of *An. coluzzii* and *An. gambiae* s.s. using the primers F6.1a (5′-TCGCCTTAGACCTTGCGTTA-3′) and R6.1b (5′-CGCTTCAAGAATTCGAGATAC-3′), respectively [40]. The PCR reactions were performed in a 25 μl reaction, which contained 0.4 μM of each primer. The other reagents included 2× GoTaq^®^ Green Master Mix (Promega, Madison, WI, USA) 12.5 μl, and 4 μl of DNA template extracted from individual mosquitoes. The volume was adjusted with DNase-free water (6.5 μl). The amplifications were performed in a thermocycler (Alpha Cycler, UK) and programmed as follows: 94 °C for 5 min followed by 35 cycles of 94 °C for 30 s, 59 °C for 30 s, and 72 °C for 1 min. At the end of amplification, the mixture was subjected to a final extension at 72 °C for 10 min. The PCR products were allowed to migrate on 2% agarose gels stained with gel red. The species' expected band profile was 249 base pairs (bp) for *An. gambiae* s.s., 479 bp for *An. coluzzii*, and 249, 479 bp for hybrid (*An. gambiae* M and S forms) after visualization with a BioDoc-it imaging system (UVP, Upland, CA, USA).

### WHO insecticide susceptibility tests

Two pyrethroid insecticides impregnated papers (0.75% permethrin and 0.05% deltamethrin) obtained from Universiti Sains Malaysia, Malaysia, were used to test the susceptibility of *An. gambiae* s.l. mosquitoes (both Tiassale and Kisumu strains) raised at 25, 28, 30, 32, and 34 °C. Permethrin and deltamethrin insecticides were selected because they are the most common insecticides used in malaria control programs [41]. The tests were performed at a constant temperature of 27 ± 2 °C following the standard WHO protocol [4].

For each mosquito strain, 20–25 female mosquitoes (aged 3–5 days), which were not fed a blood meal were aspirated into six plain paper-lined WHO holding tubes; two tubes labeled as control replicates and four as insecticide-exposed replicates [4] and observed under each temperature regime. Mosquitoes were gently placed into exposure tubes and observed for 1 h. The knockdown time (KDT) of females was recorded at 10, 15, 20, 30, 40, 50, and 60 min exposure period. After exposure, mosquitoes were transferred into holding tubes and provided with cotton wool soaked in 10% sugar solution. Mortality was recorded 24 h post-exposure [4].

### Biochemical analysis

The levels of four metabolic enzymes (mixed-function oxidase [MFO], GST, alpha- and beta-esterase) were measured in mosquitoes reared at different temperature regimes (25, 28, 30, 32, and 34 °C) and not exposed to insecticides. In addition, the enzyme levels were measured in mosquitoes reared at temperature regimes of 25, 28, 30, and 32 °C who survived exposure to pyrethroid insecticides (deltamethrin and permethrin). For measurement of enzyme levels of individual adult female *An. gambiae* s.l. (Tiassalé), biochemical assays were performed using the microplate enzyme system as described by Hemingway and Brogdon [42] with minor modifications. Briefly, the mosquitoes were frozen in Eppendorf tubes at −80 °C in the laboratory until analysis. Mosquitoes reared under each temperature regime (25, 28, 30, 32, and 34 °C) were individually homogenized in 1500 µl of potassium phosphate buffer (on ice). The homogenate was centrifuged at 14,000 rpm at 4 °C for 1 min, and the supernatant was used as an enzyme source for all enzyme assays. All assays were done in duplicates using 96-well microplates. The absorbance values were measured using the SpectraMax 340PC (Molecular Devices, Sunnyvale, CA, USA). The number of *An. gambiae* s.l. mosquitoes from each rearing temperature regime used for biochemical analysis of metabolic enzyme level is presented in Table 1. The assays for the four enzymes are explained as described below.

**Table 1:** Number of *An. gambiae* s.l. mosquitoes from each rearing temperature regime used for biochemical analysis of metabolic enzyme level

Temperature regime (°C)	Mosquitoes that were not exposed to pyrethroids	Mosquitoes that were exposed to pyrethroids
25	50	40
28	50	16
30	50	28
32	50	40
34	40	–

NB: Few mosquitoes survived after exposure to pyrethroid insecticides, resulting in an unequal number of mosquitoes in the pyrethroid-exposed group; all mosquitoes exposed to insecticides at 34 °C died; hence there were no live insecticide-exposed mosquitoes to test from the 34 °C temperature regime

### Mixed-function oxidase

Duplicate wells containing 100 µl of supernatant were prepared, and 200 µl of 3,3,5,5-tetramethylbenzidine (TMBZ) (with methanol as the solvent), and 25 µl of 3% hydrogen peroxide (H_2_O_2_) was added. The mixture was incubated for 5 min, and the optical density (OD) value was measured at 620 nm. The MFO level was estimated using the standard curve of absorbance for known cytochrome C concentrations. The enzyme level was expressed as equivalent units per mole of cytochrome P450/min/mg protein.

### GST assay

First, duplicate plates of 100 µl of supernatant were prepared, and 100 µl of 1-chloro-2,4-dinitrobenzene (cDNB) and reduced glutathione were added one after the other. The OD values were read immediately after adding the GST solution at 340 nm. The plate was incubated for 5 min, and the OD values were measured again at the same wavelength. Beer's law (*A* = *ε*lc) was used to calculate GST level, expressed as a mole of cDNB/min/mg protein. With the extinction coefficient (*ε*) of 4.39 mM^−1^ cm^−1^, the OD values (absorbance) were converted into mole cDNB conjugates. The path length was 0.94 cm.

### Non-specific esterase (NSE) assay

Mosquito homogenates were prepared and the supernatant poured into duplicate wells of 100 µl. To one set of the wells, 100 µl of 30 mM α-naphthyl acetate was added, while to the other, 100 µl of 30 mM β-naphthyl acetate was added. The plate was incubated at room temperature for 10 min. After incubation, 100 µl of dianisidine was added to each well, and the mixture was allowed to incubate for another 2 min, after which the OD values were measured at 620 nm. The esterase level for each substrate was calculated based on the standard curves of absorbance for known concentrations of α-naphthol or β-naphthol. The enzyme level was expressed as moles of α-naphthol or β-naphthol/min/mg protein.

### Protein assay

Because of size differences in individual mosquitoes, correction in the analyses of all enzyme levels was performed using the protein concentration as a standard correction factor. A commercial protein assay kit (Pierce Coomassie Plus, Thermo Scientific, USA) was used to obtain the bovine serum albumin standard curve. After that, the protein concentration was transformed and calculated based on the same curve. The protein assay was conducted by mixing 200 µl of Coomassie Plus dye reagent with 20 µl of mosquito homogenate and 80 µl of the potassium phosphate (K_3_PO_4_) buffer, and the plate was read at 620 nm.

### Statistical analysis

Insecticide susceptibility data were interpreted following WHO criteria: mosquitoes were defined as susceptible if mortality was greater than 98%; mortality between 90 and 98% indicated suspected resistance with more investigations needed; and mortality less than 90% suggested confirmation of the existence of resistance genes [4]. Some of the controls had mortality between 5 and 20%; therefore, mortality was corrected using Abbott's formula [43] as follows:

$${\text{Corrected}}\,{\text{mortality}}(\% ) = \frac{{{\text{Mortality\,in\,treatment}}\left( \% \right) - {\text{Mortality\,in\,control}}(\% )}}{{100 - {\text{Mortality\,in\,control}}(\% )}} \times 100$$

Probit analysis was also used to estimate KDT_50_ and mosquito's susceptibility status to permethrin and deltamethrin insecticides. The KDT_50_ is the time at which 50% of the mosquitoes were knocked down. Using IBM SPSS Statistics software (version 23.0), the number of knocked-down mosquitoes was considered the response frequency, the total number of mosquitoes per test was regarded as the total number observed, and time was considered a covariate. Log base 10 response was calculated from the data [44]. The KDT_50_ was used to calculate the knockdown resistance ratio (KRR_50_) by dividing the KDT_50_ of the test mosquitoes (*An. gambiae* s.l. Tiassalé strain) by that of the *An. gambiae* s.l. Kisumu reference susceptible strain in all four temperature regimes [45, 46]. The KRR_50_ was scaled as follows: KRR_50_ < 1 = susceptible, 1–10 = low resistance, 11–30 = moderate resistance, 31–100 = high resistance, and KRR_50_ > 100 = very high resistance [47].

With regard to metabolic enzyme data, the assumptions of normality and homogeneity of variances were assessed using Shapiro–Wilk and Bartlett's tests, respectively, in Stata version 15.1 (StataCorp LLC, TX, USA). The levels of MFO, GSTs, and NSE (alpha and beta) enzymes were not normally distributed and were analyzed using the non-parametric Kruskal–Wallis *H*-test. In cases where the overall model showed statistically significant differences, Dunn's multiple range tests were further used to determine where the differences existed. A Mann–Whitney *U*-test was used to compare the enzyme levels in mosquitoes that were not exposed and those that were exposed to pyrethroids. Unless otherwise stated, in all statistical analyses, a *P*-value of less than 0.05 was considered significant.

## Results

### Composition of *An. gambiae* s.l. (Tiassalé strain) mosquitoes

Species identification showed that *An. gambiae* s.l. samples used in the study consisted of two species; *An. gambiae* s.s. (26.53%) and *An. coluzzii* (23.47%). A hybrid of the two species constituted 50.00% (Table 2).

**Table 2:** Composition of *An. gambiae* s.l. mosquitoes

*An. gambiae* s.l.	Frequency (*N*)	Percentage (%)
*An. gambiae* s.s. (S form)	26	26.53
*An. coluzzii* (M form)	23	23.47
M/S hybrids	49	50.00
Total	98	100.00

### WHO insecticide susceptibility

#### Mortality in *An. gambiae* s.l. mosquitoes after exposure to pyrethroids

**Fig. 1:**
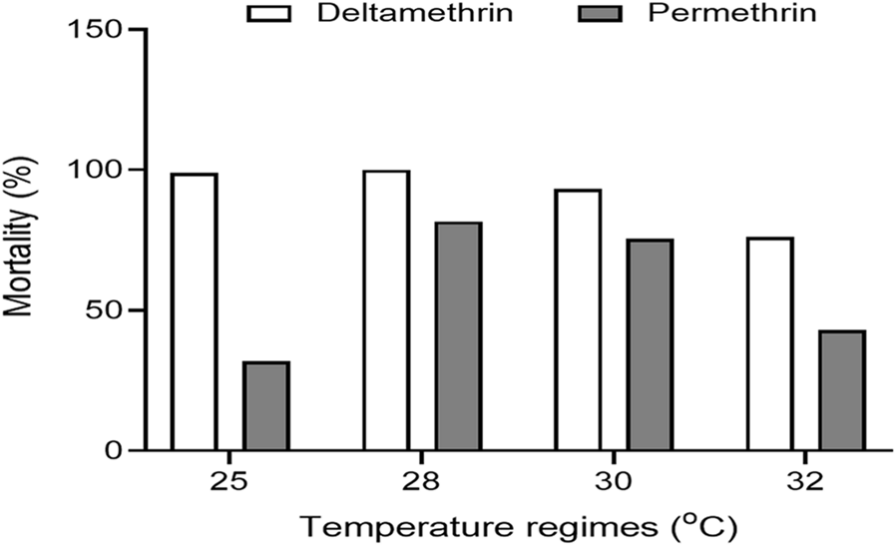
Insecticide susceptibility of *An. gambiae* s.l. (Tiassalé strain) mosquitoes reared at different temperature regimes

The results of insecticide susceptibility of *An. gambiae* s.l. (Tiassalé) to deltamethrin and permethrin insecticides are presented in Fig. 1. Adult mosquitoes that emerged at 36 °C died within the first 24 h post-emergence, and no adults emerged at 38 or 40 °C. Therefore, bioassay and molecular tests were restricted to only adults that emerged from 25, 28, 30, 32, and 34 °C. Mortality in the control replicates at 32 °C exceeded 5%; therefore, mortality was corrected, consistent with prescribed guidelines using Abbott's formula [43].

Overall, deltamethrin insecticides induced higher mortality compared with permethrin insecticides irrespective of the temperature regime. Female mosquitoes showed high mortality (100%) to deltamethrin at 28 °C and mortality decreased at temperature above 28 °C (Fig. 1). Upon exposure to permethrin, mortality in *An. gambiae* s.l. (Tiassalé) mosquitoes decreased with increasing temperature from 28 (81.61%) to 32 °C (43.06%). All susceptible *An. gambiae* s.l. (Kisumu) mosquitoes exposed to permethrin and deltamethrin insecticides died irrespective of the rearing temperature (Additional file 1:Table S2).

#### Knockdown resistance ratio

The time at which 50% of the mosquitoes were knocked down (KDT_50_) after exposure to insecticides was assessed under different temperature regimes, 25, 28, 30, 32, and 34 °C. The KDT_50_ of *An. gambiae* s.l. (Tiassalé) mosquitoes exposed to both permethrin and deltamethrin insecticides decreased with increasing temperature from 25 to 34 °C. For mosquitoes raised at 25 °C and exposed to permethrin insecticides, the KDT_50_ was higher (888.70 min) than in the other temperature regimes (Table 3). Furthermore, the KRR based on KDT_50_ for deltamethrin decreased with increasing temperature from 25 to 32 °C. With permethrin, the resistance ratio was highest at 25 °C (51.31), followed by 32 °C (4.12), 28 °C (3.87), and 30 °C (3.59) (Table 3). Generally, all the mosquitoes in this study had developed a certain level of resistance to permethrin and deltamethrin insecticides, which enabled them to survive the knockdown effect for some time.

**Table 3:** Knockdown resistance ratio of *An. gambiae* s.l. mosquitoes (Tiassalé strain) at different rearing temperature regimes

Insecticide	Temperature regime (°C)	Test population (Tiassalé) KDT_50_ [95% CI]	Reference strain (Kisumu) KDT_50_ [95% CI]	KRR	Resistance status
Deltamethrin	25	40.18 [38.22, 42.23]	16.02 [15.12, 16.94]	2.51	Low resistance
	28	32.33 [30.76, 33.88]	16.35 [15.45, 17.27]	1.98	Low resistance
	30	28.02 [24.84, 31.36]	15.68 [14.63, 16.71]	1.79	Low resistance
	32	22.85 [21.37, 24.36]	15.44 [14.33, 16.52]	1.48	Low resistance
Permethrin	25	888.70 [224.30, 7,113,093.45]	17.32 [15.83, 17.92]	51.31	High resistance
	28	63.26 [56.06, 74.90]	16.34 [15.47, 17.23]	3.87	Low resistance
	30	60.57 [55.03, 69.18]	16.88 [17.71, 20.26]	3.59	Low resistance
	32	59.29 [52.81, 69.25]	14.39 [13.47, 15.29]	4.12	Low resistance

*KDT*  knockdown time, *KRR*  knockdown resistance ratio, *CI*  confidence interval, KDT_50_ and 95% CI were obtained using Probit analysis; KRR_50_ < 1 = susceptible, 1–10 = low resistance, 11–30 = moderate resistance, 31–100 = high resistance, and KRR_50_ > 100 = very high resistance

### Influence of temperature and insecticide on the expression of metabolic enzymes

#### MFO level

**Fig. 2:**
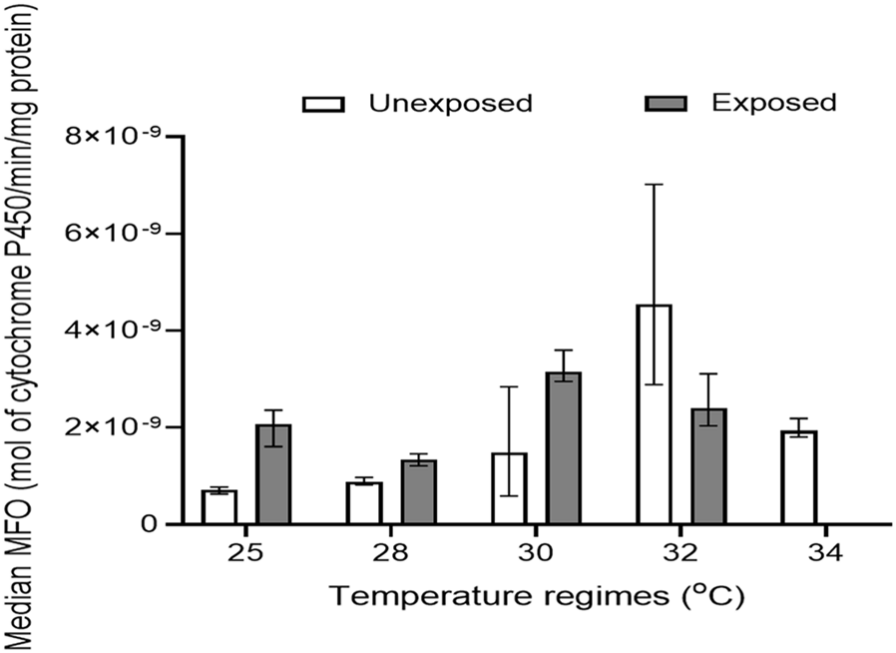
Median MFO level in *An. gambiae* s.l. mosquitoes reared at different temperature regimes. NB: no mosquito reared at 34 °C survived after being exposed to pyrethroids; hence, no enzyme level was measured

The level of MFO was assessed in mosquitoes reared at 25, 28, 30, 32, and 34 °C and not exposed to pyrethroids. The results showed that the MFO level was more elevated in mosquitoes reared at 32 °C [4.55 × 10^–9^ (IQR, 4.13 × 10^–9^) mol cytochrome P450/min/mg protein] compared to those reared at 34 °C [1.94 × 10^–9^ (IQR, 3.80 × 10^–10^)], 30 °C [1.49 × 10^–9^ (IQR, 2.26 × 10^–9^)], 28 °C [8.85 × 10^–10^ (IQR, 1.50 × 10^–10^)], and 25 °C [7.21 × 10^–10^ (IQR, 1.43 × 10^–10^) mol cytochrome P450/min/mg protein] (Additional file 1: Table S3). The levels decreased in the order of 32 > 34 > 30 > 28 > 25 (Fig. 2). Generally, levels of oxidase increased significantly (Kruskal–Wallis *H*-test, *H* = 144.42, *df* = 4, *P* < 0.001) with increasing temperature. Dunn's multiple range test showed that all but 30 vs 28 °C (*P* = 0.113) and 34 vs 30 °C (*P* = 0.010) showed a statistically significant difference in oxidase level (Additional file 1: Table S4).

The effect of rearing temperature on MFO level was also assessed in mosquitoes that survived after being exposed to pyrethroids. Mosquitoes reared at 30 °C had higher [3.15 × 10^–9^ (IQR, 6.40 × 10^–10^) mol cytochrome P450/min/mg protein] levels compared to those reared at 32 °C [2.40 × 10^–9^ (IQR, 1.07 × 10^–9^)], 25 °C [2.07 × 10^–9^ (IQR, 7.50 × 10^–10^)], and 28 °C [1.34 × 10^–9^ (IQR, 2.50 × 10^–10^) mol cytochrome P450/min/mg protein] (Fig. 2). There was a significant difference (Kruskal–Wallis *H*-test, *H* = 68.18, *df* = 3, *P* < 0.001) in MFO levels among the different temperature regimes. Further tests using Dunn's multiple range test showed a significant difference (*P* < 0.008) in the various temperature regime comparisons (Additional file 1: Table S4).

The MFO levels in mosquitoes reared at 25 °C (Mann–Whitney *U*-test, *z* = −7.72, *P* < 0.001), 28 °C (Mann–Whitney *U*-test, *z* = −5.33, *P* < 0.001), and 30 °C (Mann–Whitney *U*-test, *z* = −4.68, *P* < 0.001) and not exposed to pyrethroids were significantly lower than those that were exposed to pyrethroids. However, mosquitoes reared at 32 °C and not exposed to pyrethroids had significantly higher (Mann–Whitney *U*-test, *z* = 5.12, *P* < *0.001*) MFO levels than those exposed to pyrethroids (Fig. 2).

#### GST level

**Fig. 3:**
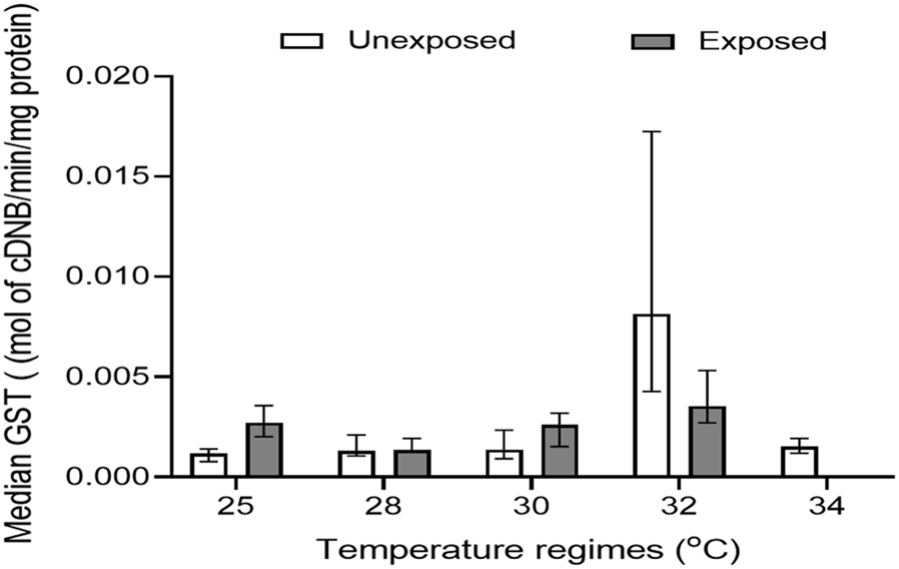
Median GST level in *An. gambiae* s.l. mosquitoes reared at different temperature regimes. NB: No mosquito reared at 34 °C survived after being exposed to pyrethroids; hence, no enzyme level was measured

The level of GST enzyme was assessed in mosquitoes reared at 25, 28, 30, 32, and 34 °C and not exposed to pyrethroids. The results showed that the GST enzyme level followed a trend (32 > 34 > 30 > 28 > 25) similar to that of the MFO level (Fig. 3). Statistically, there was a significant difference (Kruskal–Wallis H-test, *H* = 89.06, *df* = 4, *P* < 0.001) in the GST levels in mosquitoes among the different temperature regimes. However, further analysis using Dunn's multiple range test showed statistically significant differences for 32 vs 25 °C (*P* < 0.001), 34 vs 25 °C (*P* = 0.002), 32 vs 28 °C (*P* < 0.001), 32 vs 30 °C (*P* < 0.001), and 34 vs 32 °C (*P* < 0.001) (Additional file 1: Table S4).

With mosquitoes that were exposed to pyrethroids, the level of GST was highest at 32 °C [3.55 × 10^–3^ (IQR, 2.63 × 10^–3^)], followed by 25 °C [2.72 × 10^–3^ (IQR, 1.56 × 10^–3^)], 30 °C [2.62 × 10^–3^ (IQR, 1.69 × 10^–3^)] and the lowest recorded at 28 °C [1.35 × 10^–3^ (IQR, 6.59 × 10^–4^) mol cDNB/min/mg protein] (Additional file 1: Table S3). There was a statistically significant difference (Kruskal–Wallis *H*-test, *H* = 18.26, *df* = 3, *P* < 0.001) in the GST level in mosquitoes among the different temperature regimes. Further statistical tests using Dunn's multiple range test showed statistically significant differences for 28 vs 25 °C (*P* = 0.002), 32 vs 28 °C (*P* < 0.001), and 32 vs 30 °C (*P* = 0.005) (Additional file 1: Table S4). In addition, mosquitoes in the temperature regimes exposed to pyrethroids (except for those reared at 32 °C) had higher GST levels compared to those that were not exposed to pyrethroids (Fig. 3). However, all but mosquitoes reared at 28 °C (Mann–Whitney *U*-test, *z* = −0.52, *P* = 0.605) showed a statistically significant difference in GST levels (Additional file 1: Table S5).

#### Alpha-esterase level

The level of α-esterase enzyme was assessed in mosquitoes reared at 25, 28, 30, 32, and 34 °C and not exposed to pyrethroids. The highest median α-esterase level was recorded at 32 °C [1.32 × 10^–6^ (IQR, 9.41 × 10^–7^)], followed by 34 °C [4.04 × 10^–7^ (IQR, 1.56 × 10^–7^)], 28 °C [2.83 × 10^–7^ (IQR, 4.32 × 10^–7^)], 25 °C [2.52 × 10^–7^ (IQR, 7.30 × 10^–8^)] and 30 °C [2.12 × 10^–7^ (IQR, 4.10 × 10^–7^) mol α-naphthol/min/mg protein] (Additional file 1: Table S3). Median α-esterase level did not increase with increasing rearing temperature; however, there was a statistically significant difference (Kruskal–Wallis *H*-test, *H* = 99.46, *df* = 4, *P* < 0.001) in α-esterase level in mosquitoes among the different temperature regimes. Further statistical tests using Dunn's multiple range test showed a statistically significant difference for 32 vs 25 °C (*P* < 0.001), 34 vs 25 °C (*P* < 0.001), 32 vs 28 °C (*P* < 0.001), 32 vs 30 °C (*P* < 0.001), 34 vs 30 °C (*P* < 0.001), and 34 vs 32 °C (*P* < 0.001) (Additional file 1: Table S4).

**Fig. 4:**
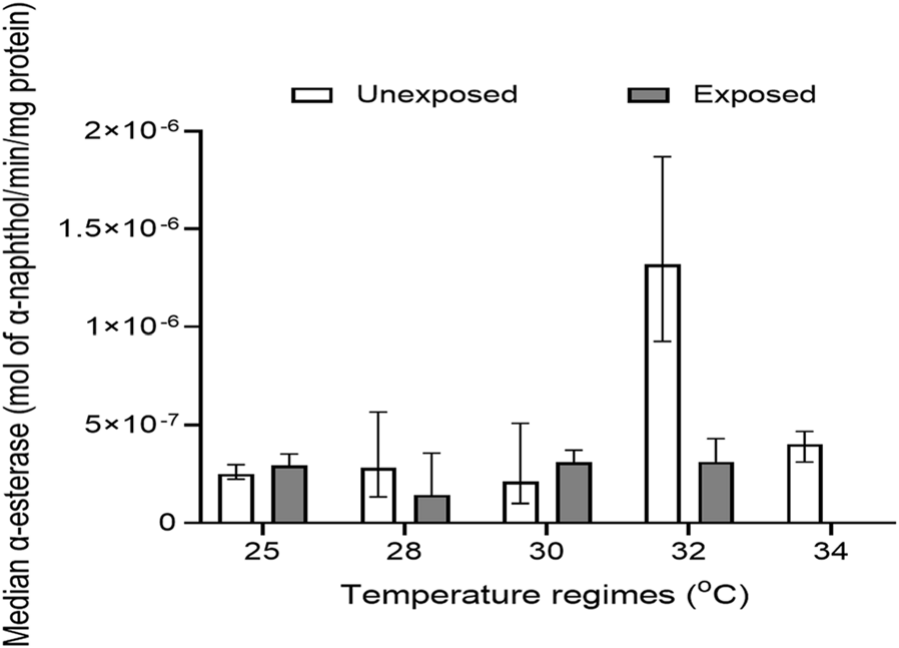
Median α-esterase level in *An. gambiae* s.l. mosquitoes reared at different temperature regimes. NB: No mosquito reared at 34 °C survived after being exposed to pyrethroids; hence, no enzyme level was measured

The level of α-esterase in mosquitoes that were exposed to pyrethroids was also assessed. The results showed that α-esterase level was highest at 32 °C [3.13 × 10^–7^ (1.81 × 10^–7^)] and lowest at 28 °C [1.44 × 10^–7^ (2.40 × 10^–7^) mol α-naphthol/min/mg protein] (Additional file 1: Table S3). There was a significant difference (Kruskal–Wallis *H*-test, *H* = 11.31, *df* = 3, *P* = 0.010) in α-esterase levels in mosquitoes among the different temperature regimes. According to Dunn's multiple range tests, a statistical difference existed only for 30 vs 28 °C (*P* = 0.003) and 32 vs 28 °C (*P* = 0.001) (Additional file 1: Table S4). Overall, mosquitoes that were not exposed to pyrethroids had higher α-esterase levels than those exposed to pyrethroids (Fig. 4). A Mann–Whitney *U*-test showed that all but mosquitoes reared at 30 °C (*z* = −1.53, *P* = 0.127) showed a statistically significant difference in α-esterase level (Additional file 1: Table S5).

#### Beta-esterase level

The level of the β-esterase enzyme was assessed in mosquitoes reared at 25, 28, 30, 32, and 34 °C and not exposed to pyrethroids. The level decreased in the order of 34 °C [3.56 × 10^–7^ (IQR, 1.54 × 10^–7^)] > 32 °C [2.87 × 10^–7^ (IQR, 3.21 × 10^–7^)] > 28 °C [1.38 × 10^–7^ (IQR, 1.82 × 10^–7^)] > 25 °C [1.36 × 10^–7^ (IQR, 6.10 × 10^–8^)] > 30 °C [1.30 × 10^–7^ (IQR, 2.93 × 10^–7^) mol β-naphthol/min/mg protein] (Additional file 1: Table S3). Generally, there was a statistically significant increase (Kruskal–Wallis *H*-test, *H* = 48.28, *df* = 4, *P* < 0.001) in β-esterase levels in mosquitoes with increasing temperature. However, post hoc analysis using Dunn's multiple range test showed a statistically significant difference for 32 vs 25 °C (*P* = 0.001), 34 vs 25 °C (*P* < 0.001), 34 vs 28 °C (*P* < 0.001), 34 vs 30 °C (*P* < 0.001), and 34 vs 32 °C (*P* = 0.003) (Additional file 1: Table S4).

**Fig. 5:**
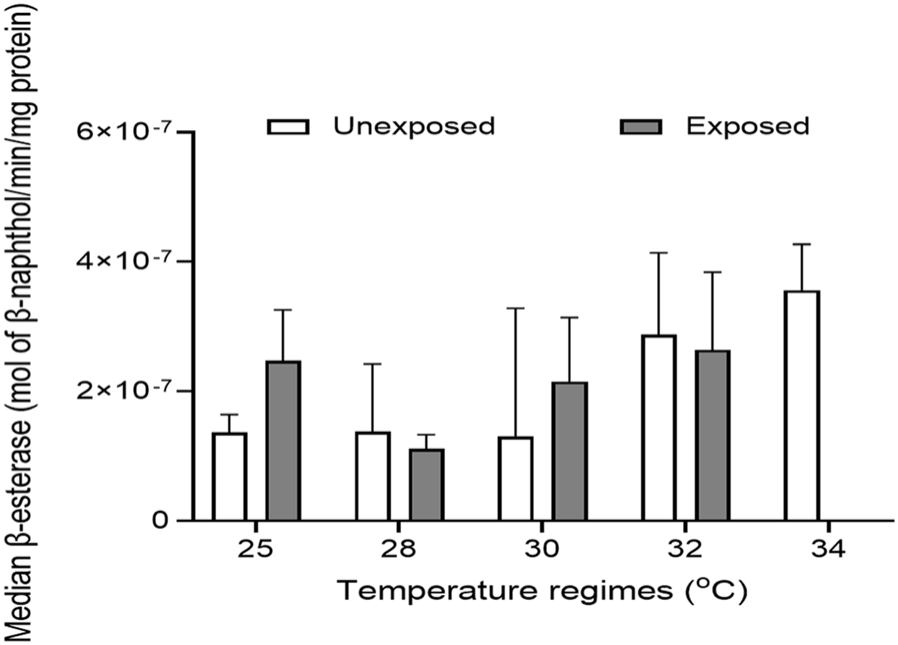
Median β-esterase level in *An. gambiae* mosquitoes reared at different temperature regimes. NB: No mosquito reared at 34 °C survived after being exposed to pyrethroids; hence, no enzyme level was measured

With regard to mosquitoes that were exposed to pyrethroids, β-esterase levels ranged from 1.11 × 10^–7^ (6.85 × 10^–8^) at 28 °C to 2.64 × 10^–7^ (1.63 × 10^–7^) mol β-naphthol/min/mg protein at 32 °C (Additional file 1: Table S3). There was a statistically significant difference (Kruskal–Wallis *H*-test, *H* = 37.91, *df* = 3, *P* = 0.001) in β-esterase levels in mosquitoes raised at different temperatures. Multiple comparisons using Dunn's test showed statistically significant differences for 28 vs 25 °C (*P* < 0.001), 30 vs 28 °C (*P* < 0.001), 32 vs 28 °C (*P* < 0.001), and 32 vs 30 °C (*P* = 0.007) (Additional file 1: Table S4). Furthermore, β-esterase levels in mosquitoes reared at 25 and 30 °C and exposed to pyrethroids were higher than in those that were not exposed to pyrethroids. On the other hand, β-esterase level in mosquitoes that were not exposed to pyrethroids was higher than in those that were raised at 28 and 32 °C and were exposed to pyrethroids (Fig. 5). However, only mosquitoes reared at 25 °C showed a statistically significant difference (Mann–Whitney *U*-test, *z* = −6.03, *P* < 0.001) in β-esterase levels (Additional file 1: Table S5).

## Discussion

Mosquito susceptibility to pyrethroids decreased with increasing temperature. This may be attributed to the higher expression of enzymes at high temperatures [48]. At high temperatures, the metabolism of insects is significantly faster [49], leading to rapid breakdown of insecticides, thereby making them relatively ineffective. This finding is in agreement with that of Oliver and Brooke [19], in which the susceptibility of *An. arabiensis* (SENN DDT strains) to pyrethroids decreased with increasing temperature. Another important finding was that deltamethrin induced higher mortality in mosquitoes than permethrin, indicating that mosquitoes were resistant to permethrin based on WHO criteria [4]. These findings are likely linked to the chemistry of the insecticides: deltamethrin belongs to the group of type II pyrethroids, which are more toxic than type I pyrethroids such as permethrin [50]. This could explain the high mortality in mosquitoes exposed to deltamethrin insecticides across all temperature regimes. This finding is in agreement with the findings of Dadzie et al. [9], which showed that *An. gambiae* s.l. mosquitoes were more susceptible to deltamethrin than permethrin. With projections of warmer temperatures in coming years, especially in sub-Saharan Africa, high resistance to pyrethroids with increasing temperatures could affect the effectiveness of malaria control programs using pyrethroids [10]. In Ghana, most mosquito control programs rely mainly on pyrethroid insecticides [51], and any future resistance will not bode well.

With regard to metabolic enzymes, MFO and GST levels in mosquitoes that were not exposed to pyrethroids increased with increasing temperature from 25 to 32 °C. On the other hand, enzyme expression levels decreased at 34 °C, suggesting that enzyme expression may be impaired above a certain optimum temperature range [52]. In addition, very high temperatures could disrupt the shape of the active site of an enzyme, hence reducing enzyme levels by preventing their formation [53]. When insects are faced with harsh conditions such as heat stress, there is an increase in the expression of enzymes such as GST and catalase in order to overcome the stress [54]. This could be a possible explanation for the increased enzyme levels at high temperatures observed in this study. These findings are corroborated by those of Kristensen and colleagues, who reported increased expression of proteasomal proteins (proteins involved in repair and degradation of oxidatively damaged proteins) in *Drosophila melanogaster* adapted to high temperatures as compared with those acclimatized to low temperatures [55]. The elevated levels of enzymes associated with higher temperatures could provide a useful understanding of the role that future warmer temperatures could play in the evolution of insecticide resistance [56].

Comparing the enzyme levels in mosquitoes that were not exposed and those exposed to pyrethroids, levels of MFO, GST, α-esterase, and β-esterase were higher in those exposed to pyrethroids than in those that were not exposed, especially at 25 and 30 °C. The high resistance of mosquitoes to insecticides might have involved metabolic detoxification due to the elevated enzyme expression in mosquitoes exposed to pyrethroids [10]. These findings are consistent with the results of Ochomo et al. [10], who observed that the levels of MFO, GST, and β-esterase in *An. gambiae* s.s. mosquitoes exposed to permethrin insecticides were higher than in mosquitoes that were not exposed to insecticides.

The study further revealed that MFO was the least expressed enzyme in mosquitoes. The findings are in agreement with those of Alemayehu et al. [2], who observed higher levels of GSTs than MFO in *An. arabiensis* mosquitoes. However, the findings of the current study are contrary to those reported by Leong et al. [25], who found more elevated levels of MFO than of α-esterase, β-esterase, and AChE in *A. aegypti* mosquitoes. These findings raise the possibility that the levels of metabolic enzymes may differ among mosquito species [57]. Therefore, understanding the relationship between temperature and expression of metabolic enzymes in a future warmer temperature regime would be of great significance for ascertaining the different metabolic enzymes involved in detoxification of insecticides.

## Conclusions

This study contributes to the knowledge of the effects of temperature variability on *An. gambiae* s.l. mosquitoes' susceptibility to insecticides and metabolic enzyme expression. The results suggest that *An. gambiae* s.l. mosquitoes reared at higher temperatures have increased insecticide resistance than those reared at lower temperatures. In addition, increased rearing temperatures of *An. gambiae* s.l. mosquitoes were associated with increased expression of metabolic enzymes. Increased metabolic enzyme levels are usually associated with insecticide resistance. This suggests that elevated temperatures projected in a future warmer climate could increase mosquitoes' resistance to insecticides and thus reduce the effectiveness of vector control measures for diseases such as malaria. Therefore, it is essential to research new tools for vector management rather than depending only on chemical insecticides.

## Supplementary Information

**Additional file 1: Table S1.** Ambient and rearing water conditions for each temperature regime. **Table S2.** Mortality in *An. gambiae* s.l. mosquitoes exposed to pyrethroids. **Table S3.** Median levels of metabolic enzymes in *An. gambiae* s.l. mosquitoes reared at different temperature regimes. **Table S4.** Pairwise comparisons of enzyme levels in *An. gambiae* s.l. mosquitoes reared at different temperature regimes. **Table S5.** Mann–Whitney *U*-test between mosquitoes that were not exposed and those exposed to pyrethroids.

## Data Availability

The data supporting the conclusions of this article are included within the article and its additional files.

## Abbreviations

AChE: Acetylcholinesterase
ARPPIS: African Regional Postgraduate Programme in Insect Science
DDT: Dichlorodiphenyltrichloroethane
GST: Glutathione S-transferase
KDT: Knockdown time
KRR: Knockdown resistance ratio
MFO: Mixed-function oxidase
NMIMR: Noguchi Memorial Institute for Medical Research
NSE: Non-specific esterase
VNVL: Vestergaard-Noguchi Memorial Institute for Medical Research Vector Labs
WHO: World Health Organization

## References

[1] Sternberg ED, Waite JL, Thomas MB (2014). Evaluating the efficacy of biological and conventional insecticides with the new ‘MCD bottle’ bioassay. Malar J.

[2] Alemayehu E, Asale A, Eba K, Getahun K, Tushune K, Bryon A (2017). Mapping insecticide resistance and characterization of resistance mechanisms in *Anopheles arabiensis* (Diptera: Culicidae) in Ethiopia. Parasites Vectors.

[3] Karunaratne S, De Silva W, Weeraratne TC, Surendran SN (2018). Insecticide resistance in mosquitoes: development, mechanisms and monitoring. Ceylon J Sci.

[4] WHO. Test procedures for insecticide resistance monitoring in malaria vector mosquitoes. World Health Organization, Geneva, Switzerland, 2nd ed. 2016. https://apps.who.int/iris/handle/10665/250677. Accessed 26 Aug 2019.

[5] Akogbéto MC, Salako AS, Dagnon F, Aikpon R, Kouletio M, Sovi A (2018). Blood feeding behaviour comparison and contribution of *Anopheles coluzzii* and *Anopheles gambiae*, two sibling species living in sympatry, to malaria transmission in Alibori and Donga region, northern Benin, West Africa. Malar J.

[6] Erlank E, Koekemoer LL, Coetzee M (2018). The importance of morphological identification of African anopheline mosquitoes (Diptera: Culicidae) for malaria control programmes. Malar J.

[7] Barrón MG, Paupy C, Rahola N, Akone-Ella O, Ngangue MF, Wilson-Bahun TA (2019). A new species in the major malaria vector complex sheds light on reticulated species evolution. Sci Rep.

[8] Gouignard N, Cherrier F, Brito-Fravallo E, Pain A, Zmarlak NM, Cailliau K (2019). Dual role of the *Anopheles coluzzii* Venus Kinase Receptor in both larval growth and immunity. Sci Rep.

[9] Dadzie SK, Chabi J, Asafu-Adjaye A, Owusu-Akrofi O, Baffoe-Wilmot A, Malm K (2017). Evaluation of piperonyl butoxide in enhancing the efficacy of pyrethroid insecticides against resistant *Anopheles gambiae* s.l. in Ghana. Malar J.

[10] Ochomo E, Bayoh M, Brogdon W, Gimnig J, Ouma C, Vulule J (2013). Pyrethroid resistance in *Anopheles gambiae* ss and *Anopheles arabiensis* in western Kenya: phenotypic, metabolic and target site characterizations of three populations. Med Vet Entomol.

[11] Shretta R, Silal SP, Celhay OJ, Gran Mercado CE, Kyaw SS, Avancena A (2019). Malaria elimination transmission and costing in the Asia-Pacific: developing an investment case. Wellcome Open Res.

[12] Recht J, Siqueira AM, Monteiro WM, Herrera SM, Herrera S, Lacerda MVG (2017). Malaria in Brazil, Colombia, Peru and Venezuela: current challenges in malaria control and elimination. Malar J.

[13] Campbell-Lendrum D, Manga L, Bagayoko M, Sommerfeld J (2015). Climate change and vector-borne diseases: what are the implications for public health research and policy?. Philos Trans R Soc B: Biol Sci.

[14] Githeko AK, Lindsay SW, Confalonieri UE, Patz JA (2000). Climate change and vector-borne diseases: a regional analysis. Bull World Health Organ.

[15] Abiodun GJ, Maharaj R, Witbooi P, Okosun KO (2016). Modelling the influence of temperature and rainfall on the population dynamics of *Anopheles arabiensis*. Malar J.

[16] Couret J, Dotson E, Benedict MQ (2014). Temperature, larval diet, and density effects on development rate and survival of *Aedes aegypti* (Diptera: Culicidae). PLoS ONE.

[17] Murdock CC, Paaijmans KP, Cox-Foster D, Read AF, Thomas MB (2012). Rethinking vector immunology: the role of environmental temperature in shaping resistance. Nat Rev Microbiol.

[18] Murdock CC, Moller-Jacobs LL, Thomas MB (2013). Complex environmental drivers of immunity and resistance in malaria mosquitoes. Proc R Soc B: Biol Sci.

[19] Oliver SV, Brooke BD (2017). The effect of elevated temperatures on the life history and insecticide resistance phenotype of the major malaria vector *Anopheles arabiensis* (Diptera: Culicidae). Malar J.

[20] Kristan M, Abeku TA, Lines J (2018). Effect of environmental variables and kdr resistance genotype on survival probability and infection rates in *Anopheles gambiae* (ss). Parasites Vectors.

[21] Whiten SR, Peterson RKD (2016). The influence of ambient temperature on the susceptibility of *Aedes aegypti* (Diptera: Culicidae) to the pyrethroid insecticide Permethrin. J Med Entomol.

[22] Yu SJ, Capinera JL (2008). Detoxification mechanisms in insects. Encyclopedia of entomology.

[23] Navarro-Roldán MA, Bosch D, Gemeno C, Siegwart M (2019). Enzymatic detoxification strategies for neurotoxic insecticides in adults of three tortricid pests. Bull Entomol Res.

[24] Abdullahi AI, Yusuf Y (2014). Response of *Anopheles gambiae* detoxification enzymes to levels of physico-chemical environmental factors from northwest Nigeria. Bayero J Pure Appl Sci.

[25] Leong C-S, Vythilingam I, Liew JW-K, Wong M-L, Wan-Yusoff WS, Lau Y-L (2019). Enzymatic and molecular characterization of insecticide resistance mechanisms in field populations of *Aedes aegypti* from Selangor, Malaysia. Parasites Vectors..

[26] Liu N (2015). Insecticide resistance in mosquitoes: impact, mechanisms, and research directions. Annu Rev Entomol.

[27] Glunt KD, Oliver SV, Hunt RH, Paaijmans KP (2018). The impact of temperature on insecticide toxicity against the malaria vectors *Anopheles arabiensis* and *Anopheles funestus*. Malar J.

[28] Glunt KD, Paaijmans KP, Read AF, Thomas MB (2014). Environmental temperatures significantly change the impact of insecticides measured using WHOPES protocols. Malar J.

[29] Agyekum TP, Botwe PK, Arko-Mensah J, Issah I, Acquah AA, Hogarh JN (2021). A systematic review of the effects of temperature on *Anopheles* mosquito development and survival: implications for malaria control in a future warmer climate. Int J Environ Res Public Health.

[30] Camara S, Koffi AA, Alou LPA, Koffi K, Kabran JPK, Koné A (2018). Mapping insecticide resistance in *Anopheles gambiae* (sl) from Côte d’Ivoire. Parasites Vectors..

[31] Qin Q, Li Y, Zhong D, Zhou N, Chang X, Li C (2014). Insecticide resistance of *Anopheles sinensis* and *An. vagus* in Hainan Island, a malaria-endemic area of China. Parasites Vectors..

[32] Asante F, Amuakwa-Mensah F (2015). Climate change and variability in Ghana: stocktaking. Climate.

[33] Agyekum TP, Arko-Mensah J, Botwe PK, Hogarh JN, Issah I, Dwomoh D (2022). Effects of elevated temperatures on the development of immature stages of *Anopheles gambiae* (s.l.) mosquitoes. Trop Med Int Health.

[34] Shapiro LLM, Whitehead SA, Thomas MB (2017). Quantifying the effects of temperature on mosquito and parasite traits that determine the transmission potential of human malaria. PLoS Biol.

[35] Edi CV, Koudou BG, Jones CM, Weetman D, Ranson H (2012). Multiple-insecticide resistance in *Anopheles gambiae* mosquitoes, Southern Côte d’Ivoire. Emerg Infect Dis.

[36] Chabi J, Van’t Hof A, N’dri LK, Datsomor A, Okyere D, Njoroge H (2019). Rapid high throughput SYBR green assay for identifying the malaria vectors *Anopheles arabiensis*, *Anopheles coluzzii* and *Anopheles gambiae *s.s. Giles. PLoS ONE.

[37] Machani MG, Ochomo E, Sang D, Bonizzoni M, Zhou G, Githeko AK (2019). Influence of blood meal and age of mosquitoes on susceptibility to pyrethroids in *Anopheles gambiae* from Western Kenya. Malar J.

[38] Mouhamadou CS, de Souza SS, Fodjo BK, Zoh MG, Bli NK, Koudou BG (2019). Evidence of insecticide resistance selection in wild *Anopheles coluzzii* mosquitoes due to agricultural pesticide use. Infect Dis Poverty.

[39] Scott JA, Brogdon WG, Collins FH (1993). Identification of single specimens of the *Anopheles gambiae* complex by the polymerase chain reaction. Am J Trop Med Hyg.

[40] Santolamazza F, Mancini E, Simard F, Qi Y, Tu Z, della Torre A (2008). Insertion polymorphisms of SINE200 retrotransposons within speciation islands of *Anopheles gambiae* molecular forms. Malar J.

[41] Monahan EA (2017). Evaluating the effectiveness of deltamethrin and permethrin in insecticide treated nets.

[42] Hemingway J, Brogdon W. Techniques to detect insecticide resistance mechanisms. 1998.

[43] Abbott WS (1987). A method of computing the effectiveness of an insecticide. J Am Mosq Control Assoc.

[44] Nnko EJ, Kihamia C, Tenu F, Premji Z, Kweka E (2017). Insecticide use pattern and phenotypic susceptibility of *Anopheles gambiae* sensu lato to commonly used insecticides in Lower Moshi, northern Tanzania. BMC Res Notes.

[45] Nwane P, Etang J, Chouaibou M, Toto JC, Kerah-Hinzoumbé C, Mimpfoundi R (2009). Trends in DDT and pyrethroid resistance in *Anopheles gambiae* s.s. populations from urban and agro-industrial settings in southern Cameroon. BMC Infect Dis.

[46] Dhiman S, Rabha B, Yadav K, Baruah I, Veer V (2014). Insecticide susceptibility and dengue vector status of wild *Stegomyia albopicta* in a strategically important area of Assam, India. Parasites Vectors..

[47] Goindin D, Delannay C, Gelasse A, Ramdini C, Gaude T, Faucon F (2017). Levels of insecticide resistance to deltamethrin, malathion, and temephos, and associated mechanisms in *Aedes aegypti* mosquitoes from the Guadeloupe and Saint Martin islands (French West Indies). Infect Dis Poverty.

[48] Matzrafi M (2019). Climate change exacerbates pest damage through reduced pesticide efficacy. Pest Manag Sci.

[49] Canola Council of Canada. Hot weather can reduce insecticide performance. 2015. https://www.canolacouncil.org/canola-watch/2015/06/10/hot-weather-can-reduce-insecticide-performance/. Accessed 29 Apr 2021.

[50] Dalefield R, Dalefield R (2017). Insecticides and acaricides. Veterinary toxicology for Australia and New Zealand.

[51] Agyekum TP. Gaseous pollutant emissions and mosquito susceptibility from the use of mosquito coils in the indoor environment. Masters Dissertation*.* Kumasi: Kwame Nkrumah University of Science and Technology; 2017.

[52] Tripathi G, Kachhwaha N, Dabi I, Bandooni N (2011). Temperature-dependent alterations in metabolic enzymes and proteins of three ecophysiologically different species of earthworms. Braz Arch Biol Technol.

[53] BBC. Factors affecting enzyme action. 2021. https://www.bbc.co.uk/bitesize/guides/zgp2v9q/revision/2. Accessed 11 Jul 2021.

[54] González-Tokman D, Córdoba-Aguilar A, Dáttilo W, Lira-Noriega A, Sánchez-Guillén RA, Villalobos F (2020). Insect responses to heat: physiological mechanisms, evolution and ecological implications in a warming world. Biol Rev.

[55] Kristensen TN, Kjeldal H, Schou MF, Nielsen JL (2016). Proteomic data reveal a physiological basis for costs and benefits associated with thermal acclimation. J Exp Biol.

[56] Polson KA, Brogdon WG, Rawlins SC, Chadee DD (2012). Impact of environmental temperatures on resistance to organophosphate insecticides in *Aedes aegypti* from Trinidad. Rev Panam Salud Pública.

[57] Farouk SA, Barahim N, Hamzah SN (2021). The detoxification enzymes activity profile in susceptible *Aedes* and *Culex* mosquitoes. IOP Conf Ser Earth Environ Sci..

