# Supplementary material for: Relationship between temperature and Anopheles gambiae sensu lato mosquitoes' susceptibility to pyrethroids and expression of metabolic enzymes
Source: Parasit Vectors. 2022 May 8;15:163. doi: 10.1186/s13071-022-05273-z (PMC9080126; doi:10.1186/s13071-022-05273-z)
Supplement: Supplementary file 1 — Additional file 1: Table S1. Ambient and rearing water conditions for each temperature regime. Table S2. Mortality in An. gambiae s.l. mosquitoes exposed to pyrethroids. Table S3. Median levels of metabolic enzymes in An. gambiae s.l. mosquitoes reared at different temperature regimes. Table S4. Pairwise comparisons of enzyme levels in An. gambiae s.l. mosquitoes reared at different temperature regimes. Table S5. Mann–Whitney U-test between mosquitoes that were not exposed and those exposed to pyrethroids. [file 13071_2022_5273_MOESM1_ESM.docx]

# Additional information

**Additional file 1: Table S1. Ambient and rearing water conditions for each temperature regime**

| **Temperature regime (°C)** | **Ambient conditions in the incubators** | | **Rearing water temperature (°C)** |
| --- | --- | --- | --- |
|  | **Temperature (°C)** | **Relative Humidity (%)** |  |
| 25 | 26.17 ± 0.30 | 84.67 ± 4.90 | 24.54 ± 0.15 |
| 28 | 27.98 ± 1.31 | 81.00 ± 4.66 | 27.41 ± 0.82 |
| 30 | 31.03 ± 0.25 | 84.05 ± 4.27 | 29.46 ± 0.14 |
| 32 | 33.05 ± 0.28 | 86.33 ± 4.54 | 30.86 ± 0.13 |
| 34 | 35.19 ± 0.50 | 84.59 ± 4.43 | 32.79 ± 0.12 |
| 36 | 36.97 ± 0.24 | 85.29 ± 3.78 | 34.79 ± 0.09 |
| 38 | 38.88 ± 0.30 | 80.40 ± 5.20 | 36.29 ± 0.10 |
| 40 | 40.95 ± 0.40 | 87.03 ± 6.62 | 38.41 ± 0.12 |

**Additional file 1: Table S2. Mortality of *An. gambiae* s.l. mosquitoes exposed to pyrethroids**

| **Mosquito strain** | **Temperature regime (^o^C)** | **Insecticide** | **Number of death** | **Total** | **Mortality (%)** |
| --- | --- | --- | --- | --- | --- |
| Tiassalé | 25 | Deltamethrin | 93 | 94 | 98.94 |
|  |  | Permethrin | 28 | 88 | 31.82 |
|  |  | Control | 0 | 42 | 0.0 |
|  | 28 | Deltamethrin | 85 | 85 | 100.00 |
|  |  | Permethrin | 71 | 87 | 81.61 |
|  |  | Control | 0 | 41 | 0.0 |
|  | 30 | Deltamethrin | 83 | 89 | 93.26 |
|  |  | Permethrin | 67 | 89 | 75.28 |
|  |  | Control | 0 | 41 | 0.0 |
|  | 32 | Deltamethrin | 75 | 95 | 78.95 |
|  |  | Permethrin | 46 | 92 | 50.00 |
|  |  | Control | 5 | 41 | 12.20* |
|  | 34 | Deltamethrin | 60 | 60 | 100.00 |
|  |  | Permethrin | 60 | 60 | 100.00 |
|  |  | Control | 18 | 41 | 43.90** |
| Kisumu | 25 | Deltamethrin | 85 | 85 | 100.00 |
|  |  | Permethrin | 92 | 92 | 100.00 |
|  |  | Control | 0 | 48 | 0.0 |
|  | 28 | Deltamethrin | 84 | 84 | 100.00 |
|  |  | Permethrin | 89 | 89 | 100.00 |
|  |  | Control | 0 | 40 | 0.0 |
|  | 30 | Deltamethrin | 81 | 81 | 100.00 |
|  |  | Permethrin | 81 | 81 | 100.00 |
|  |  | Control | 4 | 42 | 9.52 |
|  | 32 | Deltamethrin | 83 | 83 | 100.00 |
|  |  | Permethrin | 82 | 82 | 100.00 |
|  |  | Control | 3 | 46 | 6.52 |
|  | 34 | Deltamethrin | - | - | - |
|  |  | Permethrin | - | - | - |
|  |  | Control | - | - | - |

Mortality in control replicates of Tiassalé strain kept at 34 ^o^C exceeded 20 %, hence, results were excluded in the final analysis; Kisumu mosquitoes kept at 34 ^o^C failed to develop, hence no test conducted

**Additional file 1: Table S3. Median levels of metabolic enzyme in *An. gambiae* s.l.** **mosquitoes reared at different temperature regimes**

| **Status of mosquito** | **Temperature regime (°C)** | **Level of enzyme (mole/min/mg protein)** | | | |
| --- | --- | --- | --- | --- | --- |
|  |  | **MFO (IQR)** | **GST (IQR)** | **α-EST (IQR)** | **β-EST (IQR)** |
| Unexposed to pyrethroids | 25 | 7.21×10^-10^ (1.43×10^-10^) | 1.18×10^-3^ (6.25×10^-4^) | 2.52×10^-7^ (7.30×10^-8^) | 1.36×10^-7^ (6.10×10^-8^) |
|  | 28 | 8.85×10^-10^ (1.50×10^-10^) | 1.31×10^-3^ (1.05×10^-3^) | 2.83×10^-7^ (4.32×10^-7^) | 1.38×10^-7^ (1.82×10^-7^) |
|  | 30 | 1.49×10^-9^ (2.26×10^-9^) | 1.37×10^-3^ (1.43×10^-3^) | 2.12×10^-7^ (4.10×10^-7^) | 1.30×10^-7^ (2.93×10^-7^) |
|  | 32 | 4.55×10^-9^ (4.13×10^-9^) | 8.16×10^-3^ (1. 30×10^-2^) | 1.32×10^-6^ (9.41×10^-7^) | 2.87×10^-7^ (3.21×10^-7^) |
|  | 34 | 1.94×10^-9^ (3.80×10^-10^) | 1.52×10^-3^ (7.18×10^-3^) | 4.04×10^-7^ (1.56×10^-7^) | 3.56×10^-7^ (1.54×10^-7^) |
| Exposed to pyrethroids | 25 | 2.07×10^-9^ (7.50×10^-10^) | 2.72×10^-3^ (1.56×10^-3^) | 2.94×10^-7^ (1.20×10^-7^) | 2.47×10^-7^ (1.32×10^-7^) |
|  | 28 | 1.34×10^-9^ (2.50×10^-10^) | 1.35×10^-3^ (6.59×10^-4^) | 1.44×10^-7^ (2.40×10^-7^) | 1.11×10^-7^ (6.85×10^-8^) |
|  | 30 | 3.15×10^-9^ (6.40×10^-10^) | 2.62×10^-3^ (1.69×10^-3^) | 3.12×10^-7^ (1.17×10^-7^) | 2.15×10^-7^ (1.59×10^-7^) |
|  | 32 | 2.40×10^-9^ (1.07×10^-9^) | 3.55×10^-3^ (2.63×10^-3^) | 3.13×10^-7^ (1.81×10^-7^) | 2.64×10^-7^ (1.63×10^-7^) |

MFO = Mixed-Function Oxidase, GST = Glutathione-S-transferase, α-EST = α-Esterase, β-EST = β-Esterase, and IQR = Inter Quartile Range

**Additional file 1: Table S4. Pairwise comparisons of enzyme levels in *An. gambiae (s.l.)*** **mosquitoes reared at different temperature regimes**

| **Mosquito status** | **Temperature regime (^o^C)** | **MFO** | | **GST** | | **α-esterase** | | **β-esterase** | |  |
| --- | --- | --- | --- | --- | --- | --- | --- | --- | --- | --- |
|  |  | **H** | **p-value** | **H** | **p-value** | **H** | **p-value** | **H** | **p-value** |  |
| Unexposed to pyrethroids | 28 vs 25 | -3.10 | 0.001* | -2.23 | 0.013 | -1.36 | 0.087 | -0.70 | 0.241 |  |
|  | 30 vs 25 | -5.33 | < 0.001* | -2.42 | 0.008 | -0.18 | 0.430 | -0.86 | 0.194 |  |
|  | 32 vs 25 | -1.12×10^1^ | < 0.001* | -9.03 | < 0.001* | -8.56 | < 0.001* | -3.18 | 0.001* |  |
|  | 34 vs 25 | -7.37 | < 0.001* | -2.86 | 0.002* | -3.47 | < 0.001* | -6.14 | < 0.001* |  |
|  | 30 vs 28 | -2.24 | 0.013 | -0.17 | 0.431 | 1.18 | 0.120 | -0.17 | 0.433 |  |
|  | 32 vs 28 | -8.11 | < 0.001* | -6.82 | < 0.001* | -7.23 | < 0.001* | -2.44 | 0.007 |  |
|  | 34 vs 28 | -4.45 | < 0.001* | -0.73 | 0.234 | -2.18 | 0.015 | -5.30 | < 0.001* |  |
|  | 32 vs 30 | -5.90 | < 0.001* | -6.68 | < 0.001* | -8.35 | < 0.001* | -2.24 | 0.013 |  |
|  | 34 vs 30 | -2.35 | 0.010 | -0.56 | 0.286 | -3.28 | < 0.001* | -5.04 | < 0.001* |  |
|  | 34 vs 32 | 3.26 | 0.001* | 5.81 | < 0.001* | 4.69 | < 0.001* | -2.73 | 0.003* |  |
| Exposed to pyrethroids | 28 vs 25 | 3.23 | 0.001** | 2.89 | 0.002** | 2.36 | 0.009 | 4.82 | < 0.001** |  |
|  | 30 vs 25 | -5.83 | < 0.001** | 0.86 | 0.195 | -0.69 | 0.247 | 0.95 | 0.171 |  |
|  | 32 vs 25 | -2.87 | 0.002** | -1.96 | 0.025 | -1.20 | 0.115 | -1.71 | 0.044 |  |
|  | 30 vs 28 | -7.63 | < 0.001** | -2.14 | 0.016 | -2.76 | 0.003** | -3.80 | < 0.001** |  |
|  | 32 vs 28 | -5.40 | < 0.001** | -4.14 | < 0.001** | -3.27 | 0.001** | -6.08 | < 0.001** |  |
|  | 32 vs 30 | 3.23 | 0.001** | -2.59 | 0.005** | -0.40 | 0.343 | -2.48 | 0.007** |  |

H = Kruskal-Wallis test statistics; single asterisk (*) and double asterisk (**) represent significant differences at *P < 0.005* and *P < 0.008* respectively (according to Dunn multiple range test).

**Additional file 1: Table S5. Mann-Whitney U test between mosquitoes that were not exposed and those exposed to pyrethroids**

| **Metabolic enzyme** | **Temperature regime (^o^C)** | **z-value** | **p-value** |
| --- | --- | --- | --- |
| MFO | 25 | -7.72 | < 0.001* |
|  | 28 | -5.33 | < 0.001* |
|  | 30 | -4.68 | < 0.001* |
|  | 32 | 5.12 | < 0.001* |
| GST | 25 | -8.12 | < 0.001* |
|  | 28 | -0.52 | 0.605 |
|  | 30 | -2.48 | 0.013* |
|  | 32 | 3.79 | < 0.001* |
| α-esterase | 25 | -2.42 | 0.016* |
|  | 28 | 2.26 | 0.024* |
|  | 30 | -1.53 | 0.127 |
|  | 32 | 6.94 | < 0.001* |
| β-esterase | 25 | -6.03 | < 0.001* |
|  | 28 | 1.41 | 0.159 |
|  | 30 | -1.46 | 0.144 |
|  | 32 | -0.90 | 0.368 |

MFO = Mixed-Function Oxidase, GST = Glutathione-S-transferase, single asterisk (*) represents significant differences at *P < 0.05*.
